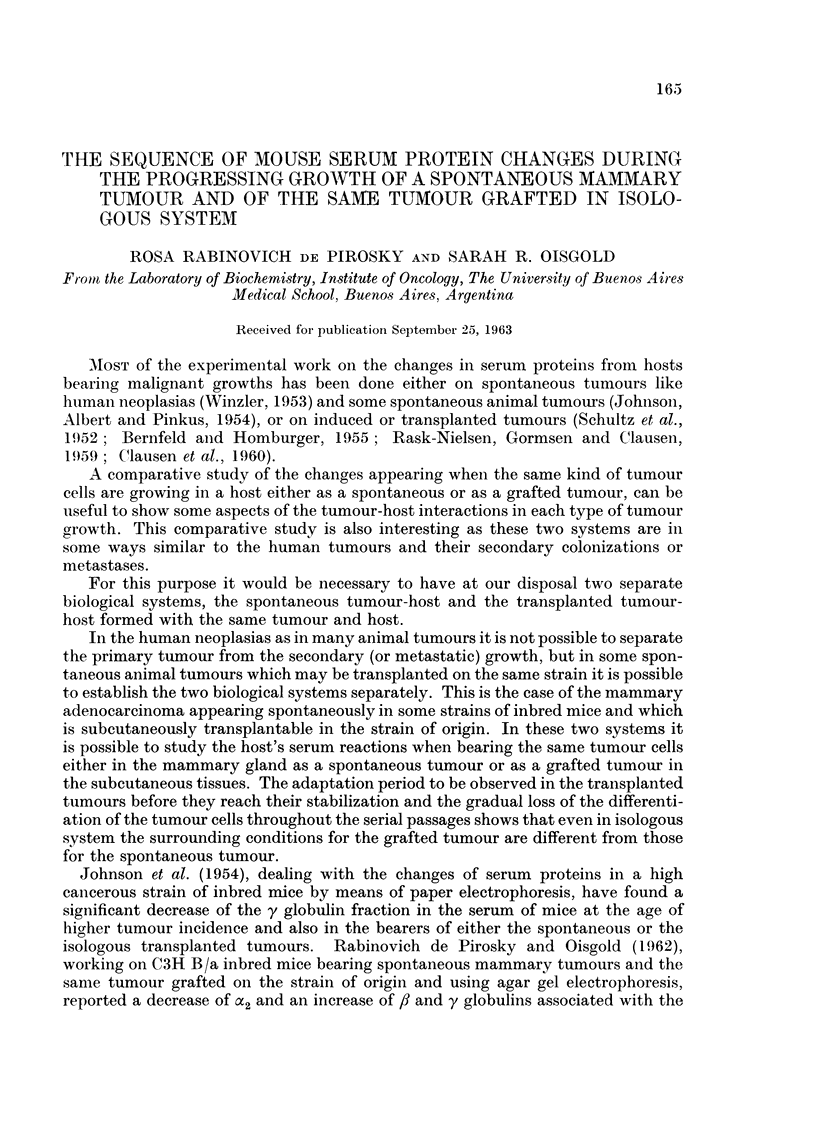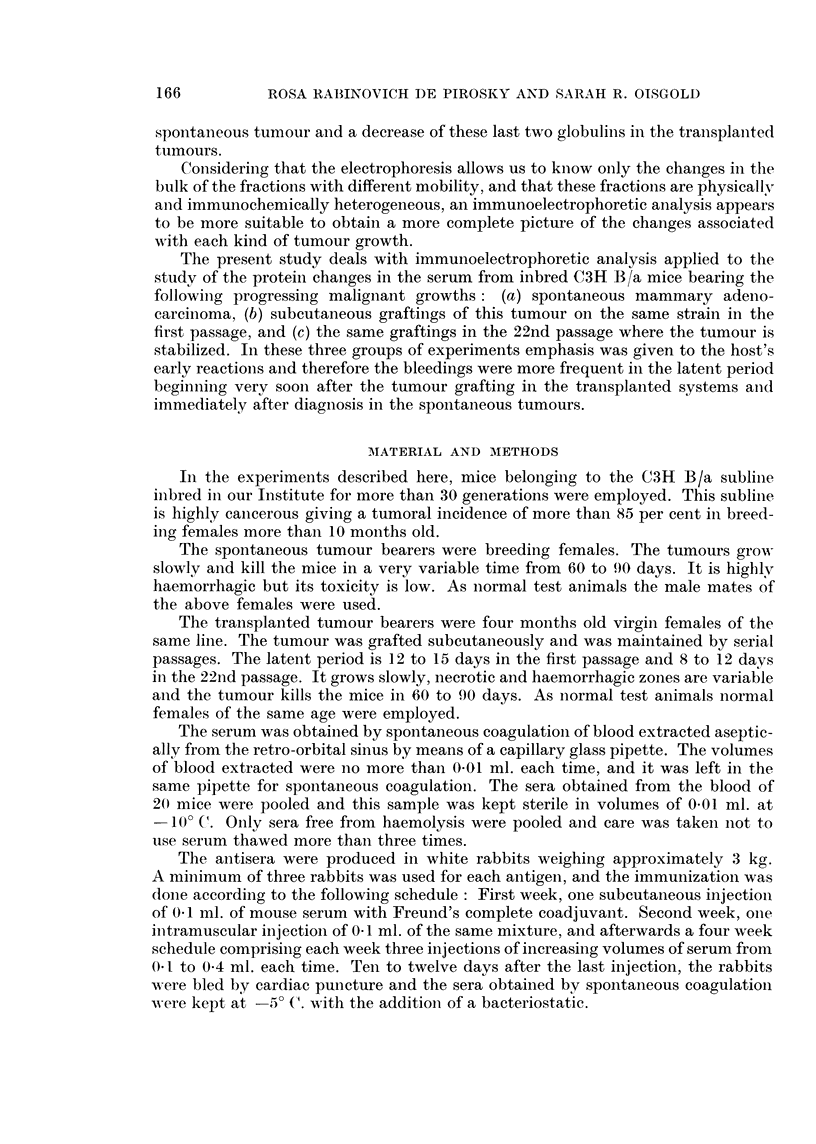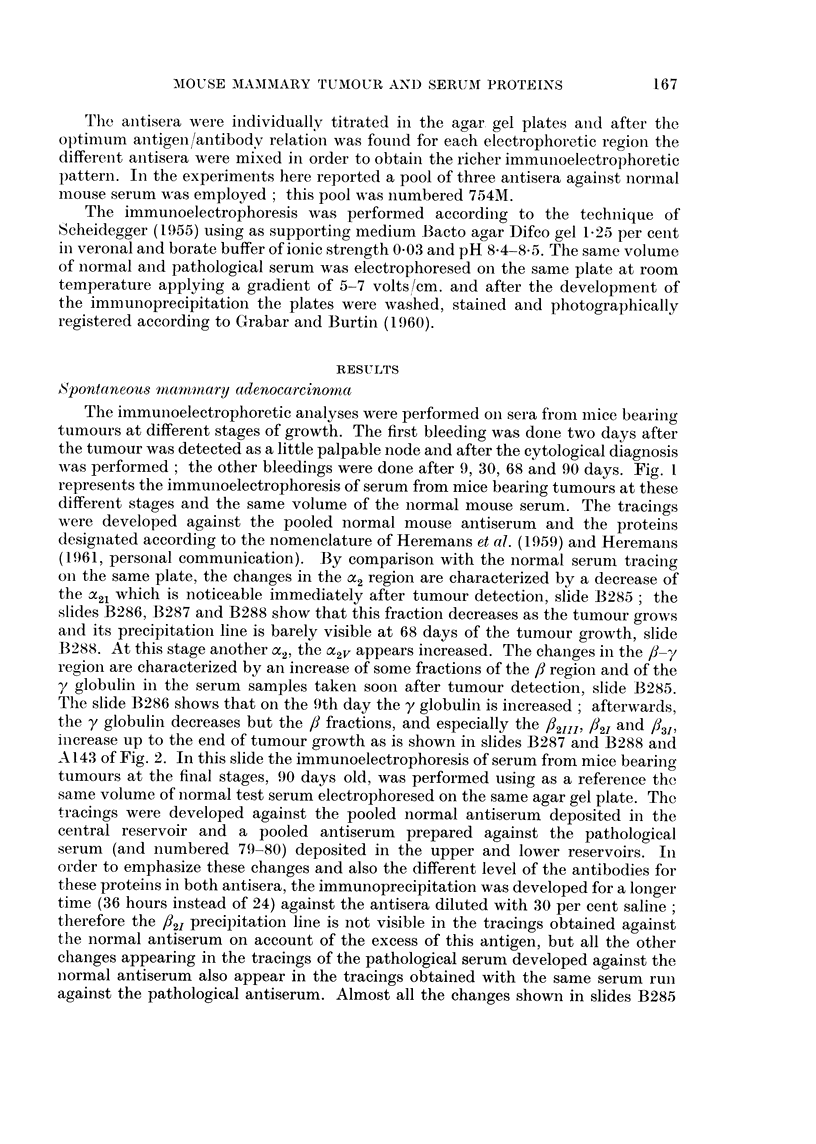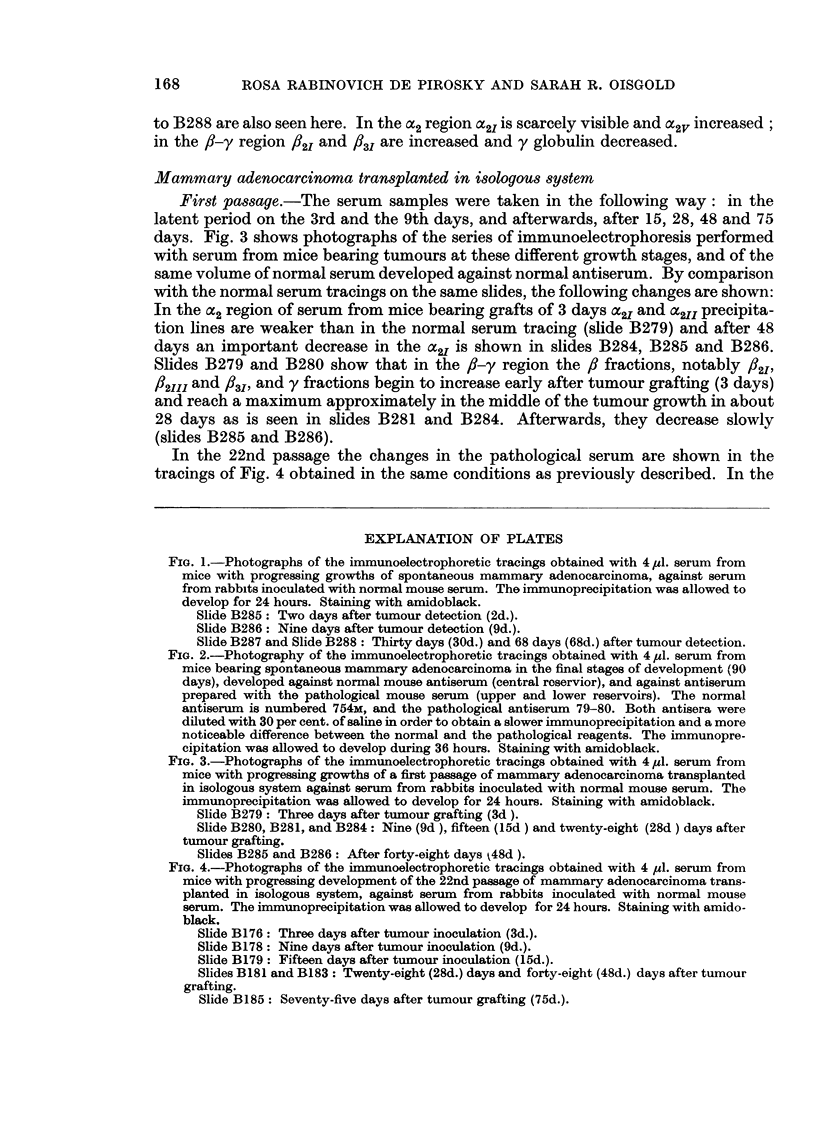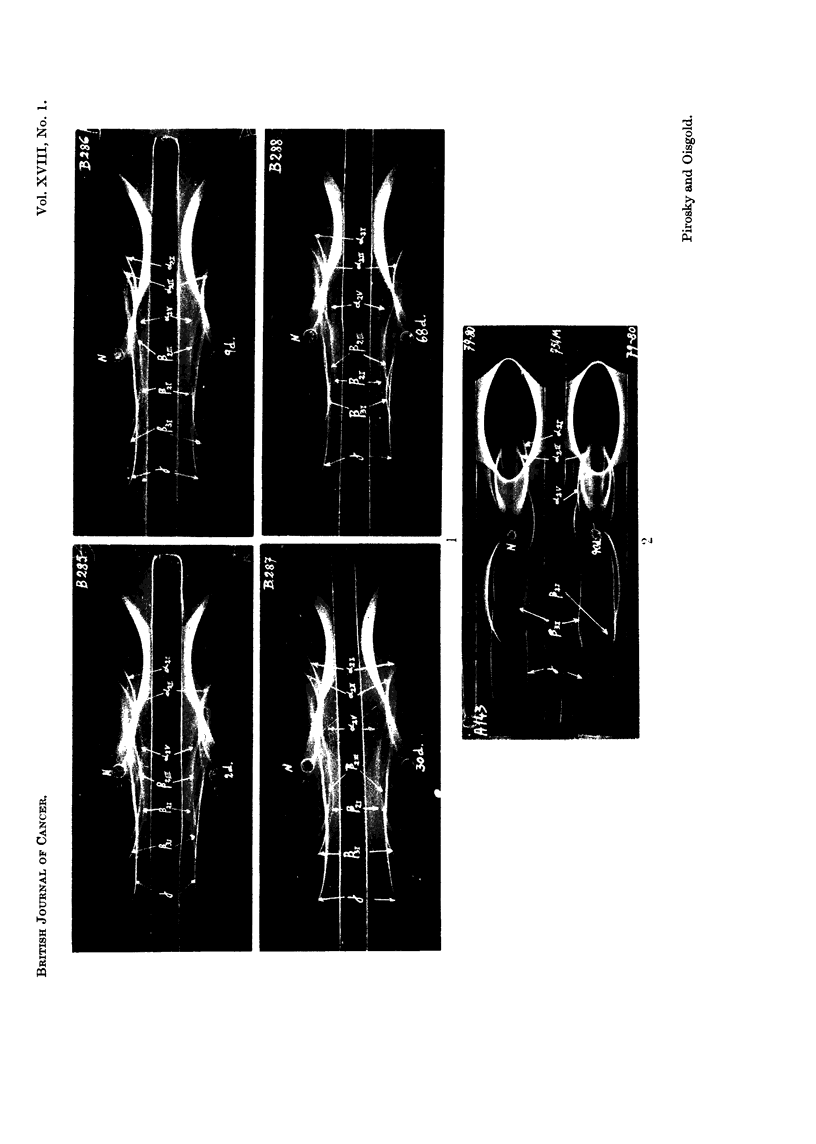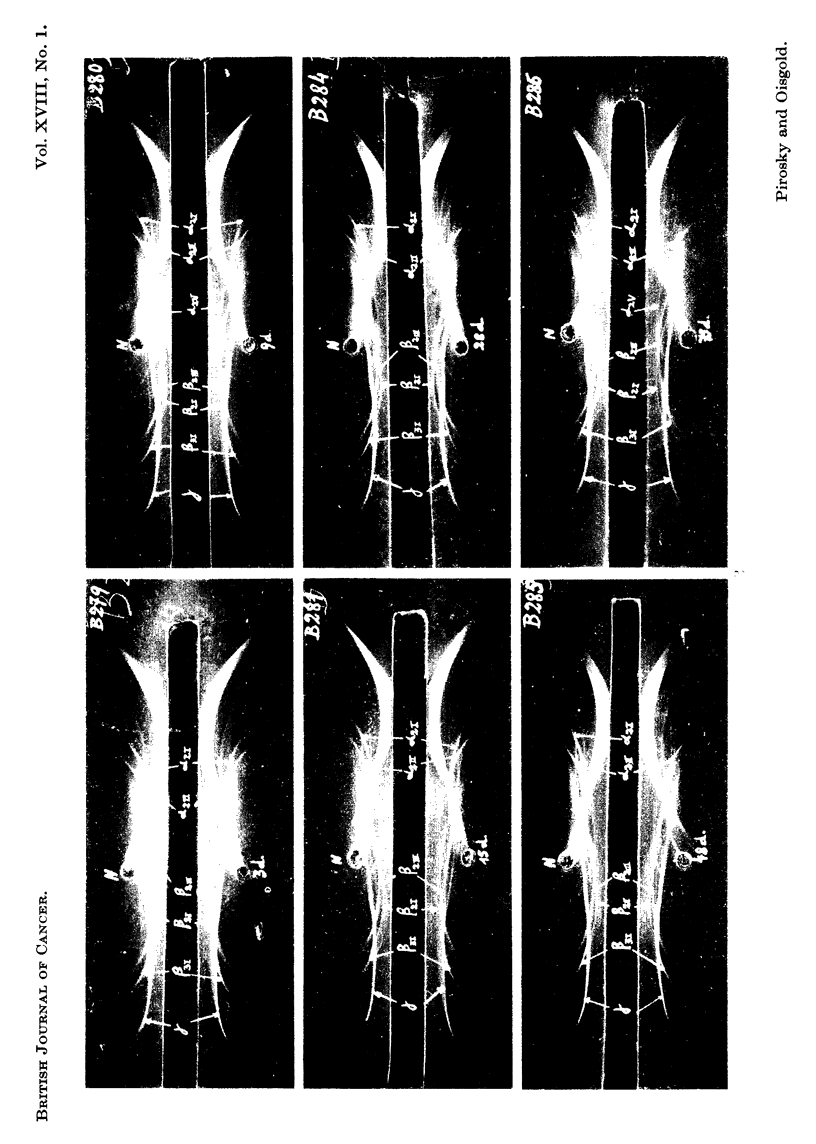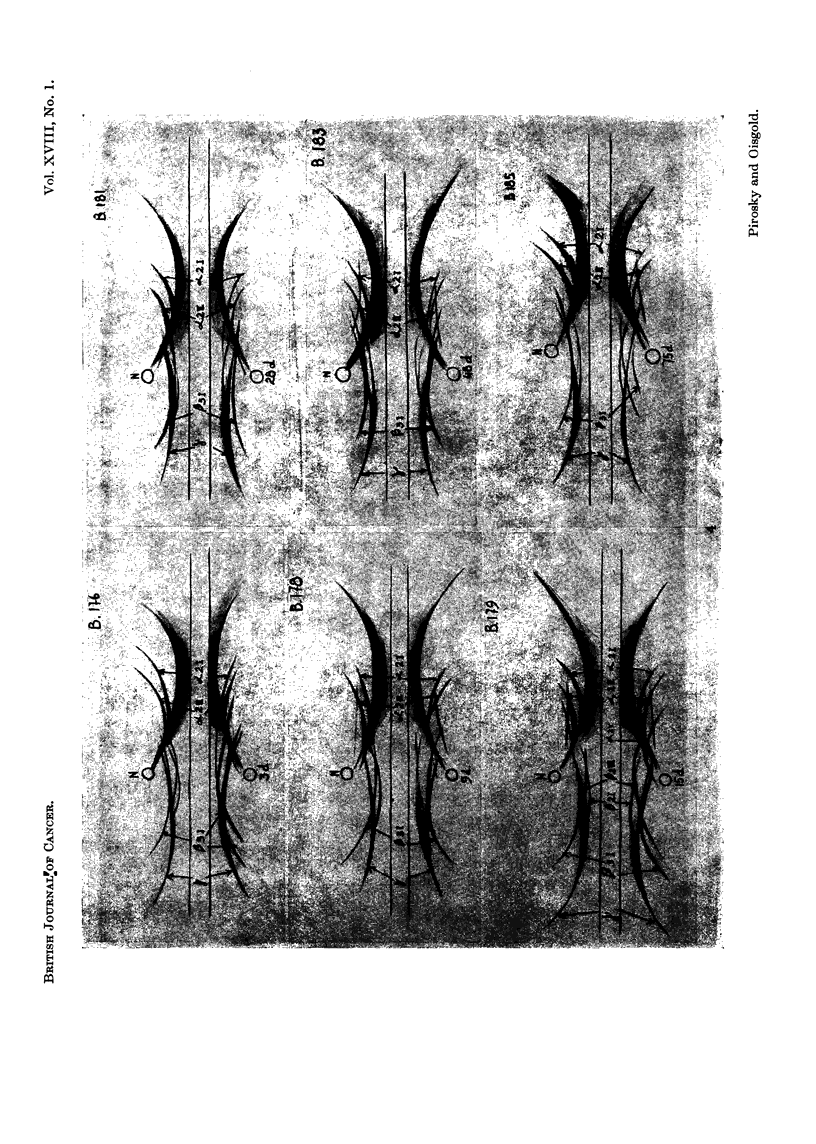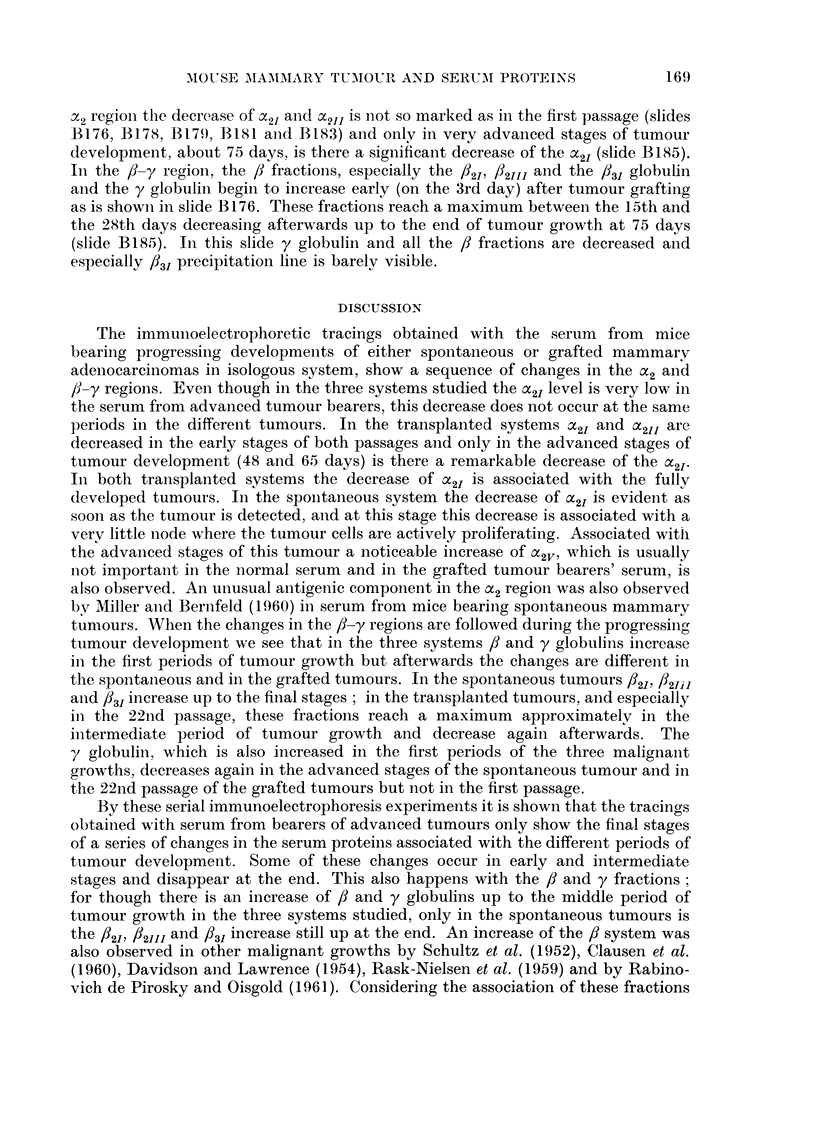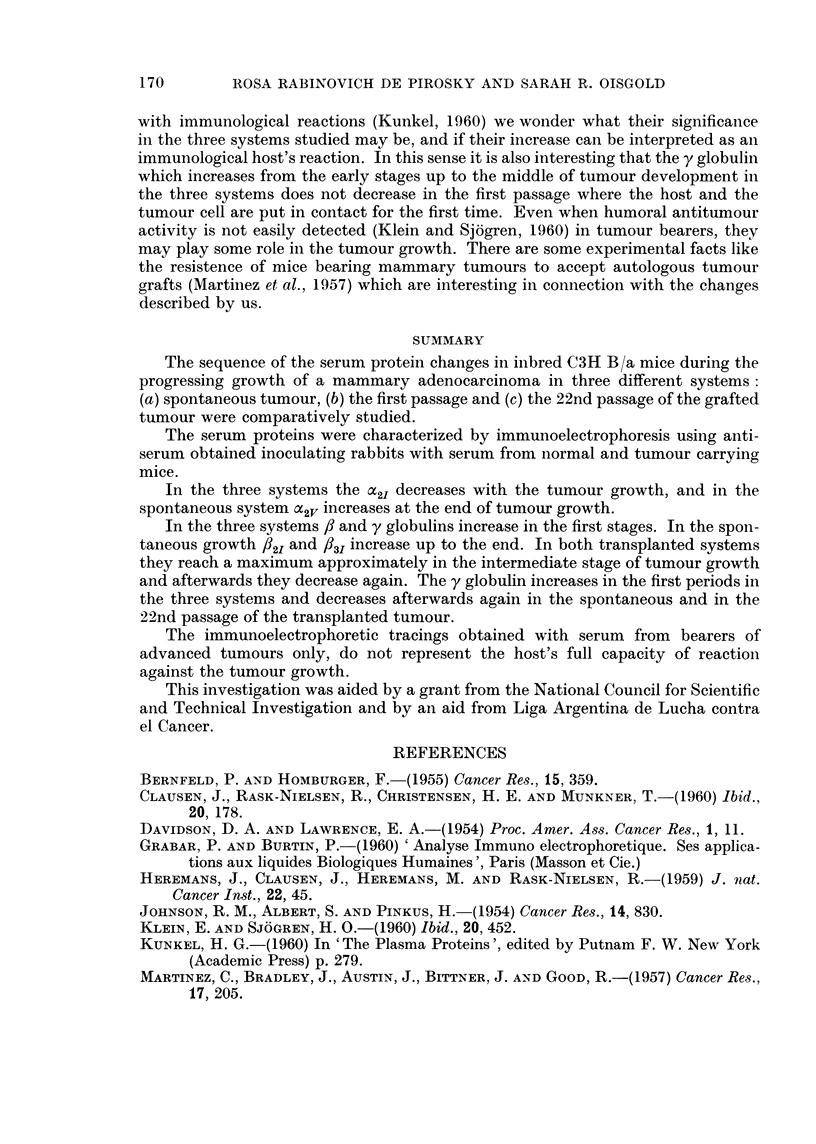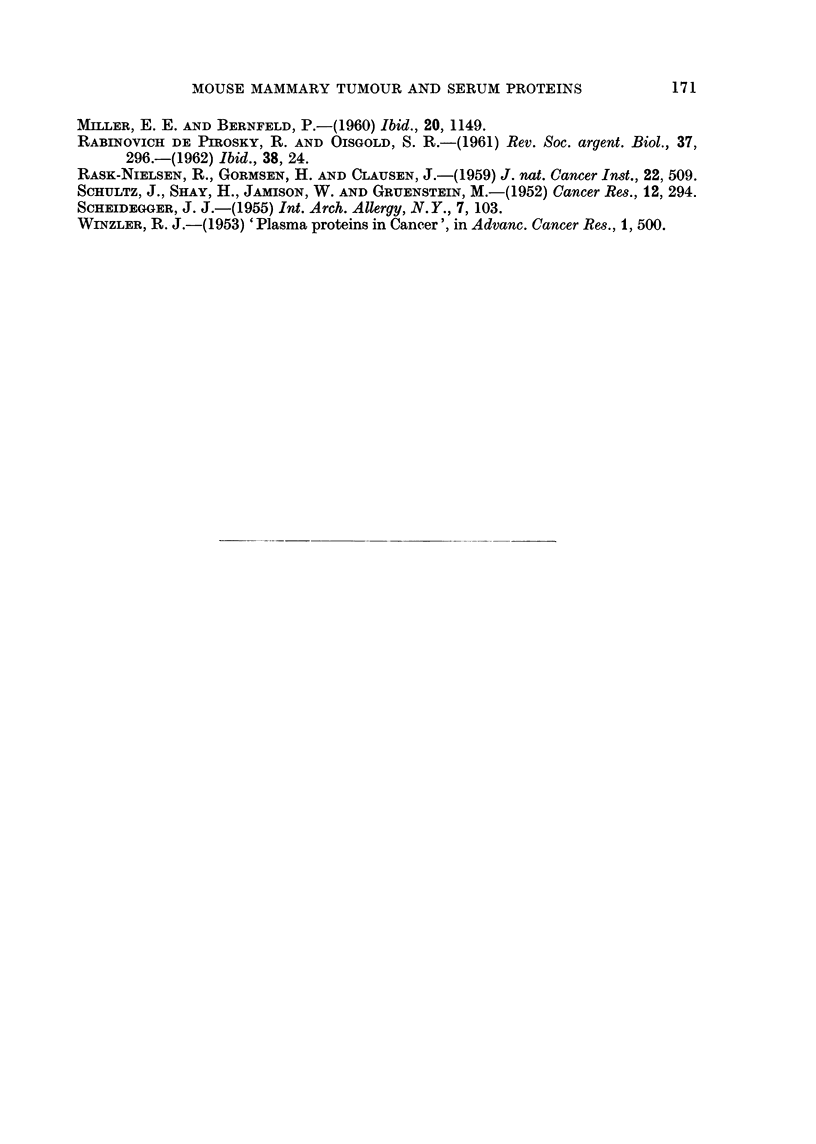# The Sequence of Mouse Serum Protein Changes During the Progressing Growth of a Spontaneous Mammary Tumour and of the Same Tumour Grafted in Isologous System

**DOI:** 10.1038/bjc.1964.19

**Published:** 1964-03

**Authors:** Rosa Rabinovich de Pirosky, Sarah R. Oisgold

## Abstract

**Images:**


### THE SEQUENCE OF MOUSE SERUM PROTEIN CHANGES DURING THE PROGRESSING GROWTH OF A SPONTANEOUS MAMMARY TUMOUR AND OF THE SAME TUMOUR GRAFTED IN ISOLOGOUS SYSTEM

ROSA RABINOVICH DE PIROSKY AND SARAH R. OISGOLD

*From the Laboratory of Biochemistry, Institute of Oncology, The University of Buenos Aires Medical School, Buenos Aires, Argentina*

Received for publication September 25, 1963

MOST of the experimental work on the changes in serum proteins from hosts bearing malignant growths has been done either on spontaneous tumours like human neoplasias (Winzler, 1953) and some spontaneous animal tumours (Johnson, Albert and Pinkus, 1954), or on induced or transplanted tumours (Schultz *et al.*, 1952 ; Bernfeld and Homburger, 1955 ; Rask-Nielsen, Gormsen and Clausen, 1959 ; Clausen *et al.*, 1960).

A comparative study of the changes appearing when the same kind of tumour cells are growing in a host either as a spontaneous or as a grafted tumour, can be useful to show some aspects of the tumour-host interactions in each type of tumour growth. This comparative study is also interesting as these two systems are in some ways similar to the human tumours and their secondary colonizations or metastases.

For this purpose it would be necessary to have at our disposal two separate biological systems, the spontaneous tumour-host and the transplanted tumour-host formed with the same tumour and host.

In the human neoplasias as in many animal tumours it is not possible to separate the primary tumour from the secondary (or metastatic) growth, but in some spontaneous animal tumours which may be transplanted on the same strain it is possible to establish the two biological systems separately. This is the case of the mammary adenocarcinoma appearing spontaneously in some strains of inbred mice and which is subcutaneously transplantable in the strain of origin. In these two systems it is possible to study the host's serum reactions when bearing the same tumour cells either in the mammary gland as a spontaneous tumour or as a grafted tumour in the subcutaneous tissues. The adaptation period to be observed in the transplanted tumours before they reach their stabilization and the gradual loss of the differentiation of the tumour cells throughout the serial passages shows that even in isologous system the surrounding conditions for the grafted tumour are different from those for the spontaneous tumour.

Johnson *et al.* (1954), dealing with the changes of serum proteins in a high cancerous strain of inbred mice by means of paper electrophoresis, have found a significant decrease of the $\gamma$ globulin fraction in the serum of mice at the age of higher tumour incidence and also in the bearers of either the spontaneous or the isologous transplanted tumours. Rabinovich de Pirosky and Oisgold (1962), working on C3H B/a inbred mice bearing spontaneous mammary tumours and the same tumour grafted on the strain of origin and using agar gel electrophoresis, reported a decrease of $\alpha_2$ and an increase of $\beta$ and $\gamma$ globulins associated with the spontaneous tumour and a decrease of these last two globulins in the transplanted tumours.

Considering that the electrophoresis allows us to know only the changes in the bulk of the fractions with different mobility, and that these fractions are physically and immunochemically heterogeneous, an immunoelectrophoretic analysis appears to be more suitable to obtain a more complete picture of the changes associated with each kind of tumour growth.

The present study deals with immunoelectrophoretic analysis applied to the study of the protein changes in the serum from inbred C3H B/a mice bearing the following progressing malignant growths : (*a*) spontaneous mammary adenocarcinoma, (*b*) subcutaneous graftings of this tumour on the same strain in the first passage, and (*c*) the same graftings in the 22nd passage where the tumour is stabilized. In these three groups of experiments emphasis was given to the host's early reactions and therefore the bleedings were more frequent in the latent period beginning very soon after the tumour grafting in the transplanted systems and immediately after diagnosis in the spontaneous tumours.

### MATERIAL AND METHODS

In the experiments described here, mice belonging to the C3H B/a subline inbred in our Institute for more than 30 generations were employed. This subline is highly cancerous giving a tumoral incidence of more than 85 per cent in breeding females more than 10 months old.

The spontaneous tumour bearers were breeding females. The tumours grow slowly and kill the mice in a very variable time from 60 to 90 days. It is highly haemorrhagic but its toxicity is low. As normal test animals the male mates of the above females were used.

The transplanted tumour bearers were four months old virgin females of the same line. The tumour was grafted subcutaneously and was maintained by serial passages. The latent period is 12 to 15 days in the first passage and 8 to 12 days in the 22nd passage. It grows slowly, necrotic and haemorrhagic zones are variable and the tumour kills the mice in 60 to 90 days. As normal test animals normal females of the same age were employed.

The serum was obtained by spontaneous coagulation of blood extracted aseptically from the retro-orbital sinus by means of a capillary glass pipette. The volumes of blood extracted were no more than 0·01 ml. each time, and it was left in the same pipette for spontaneous coagulation. The sera obtained from the blood of 20 mice were pooled and this sample was kept sterile in volumes of 0·01 ml. at —10° C. Only sera free from haemolysis were pooled and care was taken not to use serum thawed more than three times.

The antisera were produced in white rabbits weighing approximately 3 kg. A minimum of three rabbits was used for each antigen, and the immunization was done according to the following schedule : First week, one subcutaneous injection of 0·1 ml. of mouse serum with Freund's complete coadjuvant. Second week, one intramuscular injection of 0·1 ml. of the same mixture, and afterwards a four week schedule comprising each week three injections of increasing volumes of serum from 0·1 to 0·4 ml. each time. Ten to twelve days after the last injection, the rabbits were bled by cardiac puncture and the sera obtained by spontaneous coagulation were kept at —5° C. with the addition of a bacteriostatic.

The antisera were individually titrated in the agar gel plates and after the optimum antigen/antibody relation was found for each electrophoretic region the different antisera were mixed in order to obtain the richer immunoelectrophoretic pattern. In the experiments here reported a pool of three antisera against normal mouse serum was employed ; this pool was numbered 754M.

The immunoelectrophoresis was performed according to the technique of Scheidegger (1955) using as supporting medium Bacto agar Difco gel 1·25 per cent in veronal and borate buffer of ionic strength 0·03 and pH 8·4–8·5. The same volume of normal and pathological serum was electrophoresed on the same plate at room temperature applying a gradient of 5–7 volts/cm. and after the development of the immunoprecipitation the plates were washed, stained and photographically registered according to Grabar and Burtin (1960).

### RESULTS

### *Spontaneous mammary adenocarcinoma*

The immunoelectrophoretic analyses were performed on sera from mice bearing tumours at different stages of growth. The first bleeding was done two days after the tumour was detected as a little palpable node and after the cytological diagnosis was performed ; the other bleedings were done after 9, 30, 68 and 90 days. Fig. 1 represents the immunoelectrophoresis of serum from mice bearing tumours at these different stages and the same volume of the normal mouse serum. The tracings were developed against the pooled normal mouse antiserum and the proteins designated according to the nomenclature of Heremans *et al.* (1959) and Heremans (1961, personal communication). By comparison with the normal serum tracing on the same plate, the changes in the $\alpha_2$ region are characterized by a decrease of the $\alpha_{21}$ which is noticeable immediately after tumour detection, slide B285 ; the slides B286, B287 and B288 show that this fraction decreases as the tumour grows and its precipitation line is barely visible at 68 days of the tumour growth, slide B288. At this stage another $\alpha_2$, the $\alpha_{2V}$ appears increased. The changes in the $\beta$–$\gamma$ region are characterized by an increase of some fractions of the $\beta$ region and of the $\gamma$ globulin in the serum samples taken soon after tumour detection, slide B285. The slide B286 shows that on the 9th day the $\gamma$ globulin is increased ; afterwards, the $\gamma$ globulin decreases but the $\beta$ fractions, and especially the $\beta_{2III}$, $\beta_{2I}$ and $\beta_{3I}$, increase up to the end of tumour growth as is shown in slides B287 and B288 and A143 of Fig. 2. In this slide the immunoelectrophoresis of serum from mice bearing tumours at the final stages, 90 days old, was performed using as a reference the same volume of normal test serum electrophoresed on the same agar gel plate. The tracings were developed against the pooled normal antiserum deposited in the central reservoir and a pooled antiserum prepared against the pathological serum (and numbered 79–80) deposited in the upper and lower reservoirs. In order to emphasize these changes and also the different level of the antibodies for these proteins in both antisera, the immunoprecipitation was developed for a longer time (36 hours instead of 24) against the antisera diluted with 30 per cent saline ; therefore the $\beta_{2I}$ precipitation line is not visible in the tracings obtained against the normal antiserum on account of the excess of this antigen, but all the other changes appearing in the tracings of the pathological serum developed against the normal antiserum also appear in the tracings obtained with the same serum run against the pathological antiserum. Almost all the changes shown in slides B285

to B288 are also seen here. In the $\alpha_2$ region $\alpha_{2I}$ is scarcely visible and $\alpha_{2V}$ increased ; in the $\beta$–$\gamma$ region $\beta_{2I}$ and $\beta_{3I}$ are increased and $\gamma$ globulin decreased.

*Mammary adenocarcinoma transplanted in isologous system*

*First passage.*—The serum samples were taken in the following way : in the latent period on the 3rd and the 9th days, and afterwards, after 15, 28, 48 and 75 days. Fig. 3 shows photographs of the series of immunoelectrophoresis performed with serum from mice bearing tumours at these different growth stages, and of the same volume of normal serum developed against normal antiserum. By comparison with the normal serum tracings on the same slides, the following changes are shown: In the $\alpha_2$ region of serum from mice bearing grafts of 3 days $\alpha_{2I}$ and $\alpha_{2II}$ precipitation lines are weaker than in the normal serum tracing (slide B279) and after 48 days an important decrease in the $\alpha_{2I}$ is shown in slides B284, B285 and B286. Slides B279 and B280 show that in the $\beta$–$\gamma$ region the $\beta$ fractions, notably $\beta_{2I}$, $\beta_{2III}$ and $\beta_{3I}$, and $\gamma$ fractions begin to increase early after tumour grafting (3 days) and reach a maximum approximately in the middle of the tumour growth in about 28 days as is seen in slides B281 and B284. Afterwards, they decrease slowly (slides B285 and B286).

In the 22nd passage the changes in the pathological serum are shown in the tracings of Fig. 4 obtained in the same conditions as previously described. In the

---

EXPLANATION OF PLATES

FIG. 1.—Photographs of the immunoelectrophoretic tracings obtained with 4 $\mu$l. serum from mice with progressing growths of spontaneous mammary adenocarcinoma, against serum from rabbits inoculated with normal mouse serum. The immunoprecipitation was allowed to develop for 24 hours. Staining with amidoblack.

Slide B285 : Two days after tumour detection (2d.).
Slide B286 : Nine days after tumour detection (9d.).
Slide B287 and Slide B288 : Thirty days (30d.) and 68 days (68d.) after tumour detection.

FIG. 2.—Photography of the immunoelectrophoretic tracings obtained with 4 $\mu$l. serum from mice bearing spontaneous mammary adenocarcinoma in the final stages of development (90 days), developed against normal mouse antiserum (central reservior), and against antiserum prepared with the pathological mouse serum (upper and lower reservoirs). The normal antiserum is numbered 754M, and the pathological antiserum 79–80. Both antisera were diluted with 30 per cent. of saline in order to obtain a slower immunoprecipitation and a more noticeable difference between the normal and the pathological reagents. The immunoprecipitation was allowed to develop during 36 hours. Staining with amidoblack.

FIG. 3.—Photographs of the immunoelectrophoretic tracings obtained with 4 $\mu$l. serum from mice with progressing growths of a first passage of mammary adenocarcinoma transplanted in isologous system against serum from rabbits inoculated with normal mouse serum. The immunoprecipitation was allowed to develop for 24 hours. Staining with amidoblack.

Slide B279 : Three days after tumour grafting (3d ).
Slide B280, B281, and B284 : Nine (9d ), fifteen (15d ) and twenty-eight (28d ) days after tumour grafting.
Slides B285 and B286 : After forty-eight days (48d ).

FIG. 4.—Photographs of the immunoelectrophoretic tracings obtained with 4 $\mu$l. serum from mice with progressing development of the 22nd passage of mammary adenocarcinoma transplanted in isologous system, against serum from rabbits inoculated with normal mouse serum. The immunoprecipitation was allowed to develop for 24 hours. Staining with amidoblack.

Slide B176 : Three days after tumour inoculation (3d.).
Slide B178 : Nine days after tumour inoculation (9d.).
Slide B179 : Fifteen days after tumour inoculation (15d.).
Slides B181 and B183 : Twenty-eight (28d.) days and forty-eight (48d.) days after tumour grafting.
Slide B185 : Seventy-five days after tumour grafting (75d.).

1

2

Pirosky and Oisgold.

3

Pirosky and Oisgold.

Pirosky and Oisgold.

$\alpha_2$ region the decrease of $\alpha_{2I}$ and $\alpha_{2II}$ is not so marked as in the first passage (slides B176, B178, B179, B181 and B183) and only in very advanced stages of tumour development, about 75 days, is there a significant decrease of the $\alpha_{2I}$ (slide B185). In the $\beta$–$\gamma$ region, the $\beta$ fractions, especially the $\beta_{2I}$, $\beta_{2III}$ and the $\beta_{3I}$ globulin and the $\gamma$ globulin begin to increase early (on the 3rd day) after tumour grafting as is shown in slide B176. These fractions reach a maximum between the 15th and the 28th days decreasing afterwards up to the end of tumour growth at 75 days (slide B185). In this slide $\gamma$ globulin and all the $\beta$ fractions are decreased and especially $\beta_{3I}$ precipitation line is barely visible.

### DISCUSSION

The immunoelectrophoretic tracings obtained with the serum from mice bearing progressing developments of either spontaneous or grafted mammary adenocarcinomas in isologous system, show a sequence of changes in the $\alpha_2$ and $\beta$–$\gamma$ regions. Even though in the three systems studied the $\alpha_{2I}$ level is very low in the serum from advanced tumour bearers, this decrease does not occur at the same periods in the different tumours. In the transplanted systems $\alpha_{2I}$ and $\alpha_{2II}$ are decreased in the early stages of both passages and only in the advanced stages of tumour development (48 and 65 days) is there a remarkable decrease of the $\alpha_{2I}$. In both transplanted systems the decrease of $\alpha_{2I}$ is associated with the fully developed tumours. In the spontaneous system the decrease of $\alpha_{2I}$ is evident as soon as the tumour is detected, and at this stage this decrease is associated with a very little node where the tumour cells are actively proliferating. Associated with the advanced stages of this tumour a noticeable increase of $\alpha_{2V}$, which is usually not important in the normal serum and in the grafted tumour bearers' serum, is also observed. An unusual antigenic component in the $\alpha_2$ region was also observed by Miller and Bernfeld (1960) in serum from mice bearing spontaneous mammary tumours. When the changes in the $\beta$–$\gamma$ regions are followed during the progressing tumour development we see that in the three systems $\beta$ and $\gamma$ globulins increase in the first periods of tumour growth but afterwards the changes are different in the spontaneous and in the grafted tumours. In the spontaneous tumours $\beta_{2I}$, $\beta_{2III}$ and $\beta_{3I}$ increase up to the final stages ; in the transplanted tumours, and especially in the 22nd passage, these fractions reach a maximum approximately in the intermediate period of tumour growth and decrease again afterwards. The $\gamma$ globulin, which is also increased in the first periods of the three malignant growths, decreases again in the advanced stages of the spontaneous tumour and in the 22nd passage of the grafted tumours but not in the first passage.

By these serial immunoelectrophoresis experiments it is shown that the tracings obtained with serum from bearers of advanced tumours only show the final stages of a series of changes in the serum proteins associated with the different periods of tumour development. Some of these changes occur in early and intermediate stages and disappear at the end. This also happens with the $\beta$ and $\gamma$ fractions ; for though there is an increase of $\beta$ and $\gamma$ globulins up to the middle period of tumour growth in the three systems studied, only in the spontaneous tumours is the $\beta_{2I}$, $\beta_{2III}$ and $\beta_{3I}$ increase still up at the end. An increase of the $\beta$ system was also observed in other malignant growths by Schultz *et al.* (1952), Clausen *et al.* (1960), Davidson and Lawrence (1954), Rask-Nielsen *et al.* (1959) and by Rabinovich de Pirosky and Oisgold (1961). Considering the association of these fractions with immunological reactions (Kunkel, 1960) we wonder what their significance in the three systems studied may be, and if their increase can be interpreted as an immunological host's reaction. In this sense it is also interesting that the $\gamma$ globulin which increases from the early stages up to the middle of tumour development in the three systems does not decrease in the first passage where the host and the tumour cell are put in contact for the first time. Even when humoral antitumour activity is not easily detected (Klein and Sjögren, 1960) in tumour bearers, they may play some role in the tumour growth. There are some experimental facts like the resistence of mice bearing mammary tumours to accept autologous tumour grafts (Martinez *et al.*, 1957) which are interesting in connection with the changes described by us.

### SUMMARY

The sequence of the serum protein changes in inbred C3H B/a mice during the progressing growth of a mammary adenocarcinoma in three different systems : (*a*) spontaneous tumour, (*b*) the first passage and (*c*) the 22nd passage of the grafted tumour were comparatively studied.

The serum proteins were characterized by immunoelectrophoresis using antiserum obtained inoculating rabbits with serum from normal and tumour carrying mice.

In the three systems the $\alpha_{2I}$ decreases with the tumour growth, and in the spontaneous system $\alpha_{2V}$ increases at the end of tumour growth.

In the three systems $\beta$ and $\gamma$ globulins increase in the first stages. In the spontaneous growth $\beta_{2I}$ and $\beta_{3I}$ increase up to the end. In both transplanted systems they reach a maximum approximately in the intermediate stage of tumour growth and afterwards they decrease again. The $\gamma$ globulin increases in the first periods in the three systems and decreases afterwards again in the spontaneous and in the 22nd passage of the transplanted tumour.

The immunoelectrophoretic tracings obtained with serum from bearers of advanced tumours only, do not represent the host's full capacity of reaction against the tumour growth.

This investigation was aided by a grant from the National Council for Scientific and Technical Investigation and by an aid from Liga Argentina de Lucha contra el Cancer.

### REFERENCES

Bernfeld, P. and Homburger, F.—(1955) *Cancer Res.*, **15**, 359.

Clausen, J., Rask-Nielsen, R., Christensen, H. E. and Munkner, T.—(1960) *Ibid.*, **20**, 178.

Davidson, D. A. and Lawrence, E. A.—(1954) *Proc. Amer. Ass. Cancer Res.*, **1**, 11.

Grabar, P. and Burtin, P.—(1960) ' Analyse Immuno electrophoretique. Ses applications aux liquides Biologiques Humaines ', Paris (Masson et Cie.)

Heremans, J., Clausen, J., Heremans, M. and Rask-Nielsen, R.—(1959) *J. nat. Cancer Inst.*, **22**, 45.

Johnson, R. M., Albert, S. and Pinkus, H.—(1954) *Cancer Res.*, **14**, 830.

Klein, E. and Sjögren, H. O.—(1960) *Ibid.*, **20**, 452.

Kunkel, H. G.—(1960) In 'The Plasma Proteins ', edited by Putnam F. W. New York (Academic Press) p. 279.

Martinez, C., Bradley, J., Austin, J., Bittner, J. and Good, R.—(1957) *Cancer Res.*, **17**, 205.

MILLER, E. E. AND BERNFELD, P.—(1960) *Ibid.*, **20**, 1149.
RABINOVICH DE PIROSKY, R. AND OISGOLD, S. R.—(1961) *Rev. Soc. argent. Biol.*, **37**, 296.—(1962) *Ibid.*, **38**, 24.
RASK-NIELSEN, R., GORMSEN, H. AND CLAUSEN, J.—(1959) *J. nat. Cancer Inst.*, **22**, 509.
SCHULTZ, J., SHAY, H., JAMISON, W. AND GRUENSTEIN, M.—(1952) *Cancer Res.*, **12**, 294.
SCHEIDEGGER, J. J.—(1955) *Int. Arch. Allergy, N.Y.*, **7**, 103.
WINZLER, R. J.—(1953) 'Plasma proteins in Cancer', in *Advanc. Cancer Res.*, **1**, 500.